# Basaloid follicular hamartoma associated with follicular mucinosis and inflammation^☆^

**DOI:** 10.1016/j.abd.2020.10.012

**Published:** 2021-11-14

**Authors:** Solange Edelman, Adriana Natalia Torres Huamani, María Del Valle Centeno, Andrea Bettina Cervini

**Affiliations:** Servicio de Dermatología, Hospital Nacional de Pediatría “Prof. Dr. Juan P. Garrahan”, Buenos Aires, Argentina

**Keywords:** Carcinoma, basal cell, Genes, tumor suppressor, Hamartoma

## Abstract

Basaloid follicular hamartoma is a benign, superficial malformation of hair follicles that can be mistaken both clinical and histopathologically for basal cell carcinoma. Basaloid follicular hamartoma has been linked to a mutation in the PTCH-1 gene, which is part of the same pathway involved in Gorlin-Goltz syndrome. Here we present a 9-year-old patient with an asymptomatic congenital lesion on the forehead, which increased in size over the years. Histopathology showed a basaloid follicular hamartoma associated with follicular mucinosis and inflammation. Gorlin-Goltz syndrome was ruled out by clinical examination.

## Introduction

Basaloid follicular hamartoma(BFH) is a benign lesion that should be carefully evaluated because the entity may be mistaken both clinically and histologically for basal cell carcinoma. The formation of BFH has been linked to a mutation in the Patched (PTCH-1) gene, which is part of the same pathway involved in Gorlin-Goltz syndrome.

Due to the varied clinical presentation of BFH, it is imperative to perform a skin biopsy to distinguish BFH from other common skin lesions.

Currently, there are no standard treatments for BFH.

Correct identification allows for periodic monitoring for malignant transformation while sparing patients unnecessary surgery.^1^

## Case report

A 9-year-old boy was consulted for an asymptomatic congenital lesion located on the forehead increasing in size over the years. The child was born with a cleft lip and palate which was surgically corrected. There was no family history of relevance related to this dermatosis.

**Figure 1:**
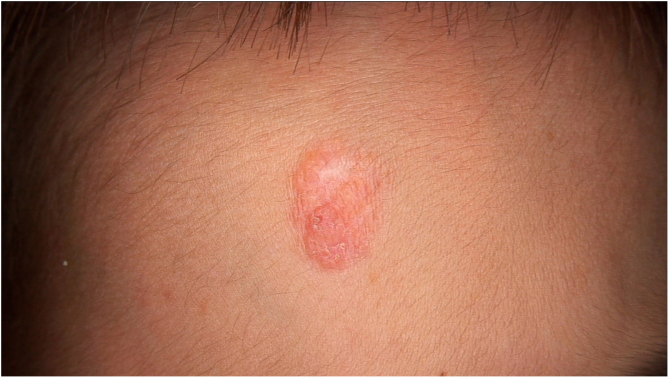
Rounded reddish plaque with warty areas.

**Figure 2:**
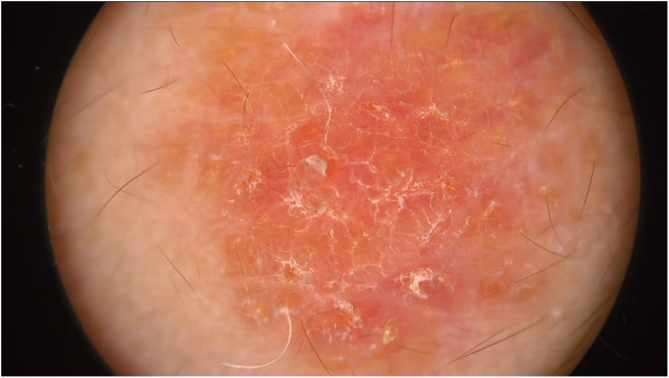
Pinkish-orange lesion with scale formation especially at the perifollicular level without a distinctive melanocytic or vascular pattern.

Physical examination showed two plantar pits and a rounded reddish plaque with warty areas in the central region of the forehead (Fig. 1). Dermoscopy revealed a reddish scaly lesion suggesting increased keratinization, mainly at the perifollicular level, without a distinctive melanocytic or vascular pattern (Fig. 2).

Based on the diagnostic suspicion of nevus sebaceous of Jadassohn or linear verrucous epidermal nevus, a biopsy was performed.

**Figure 3:**
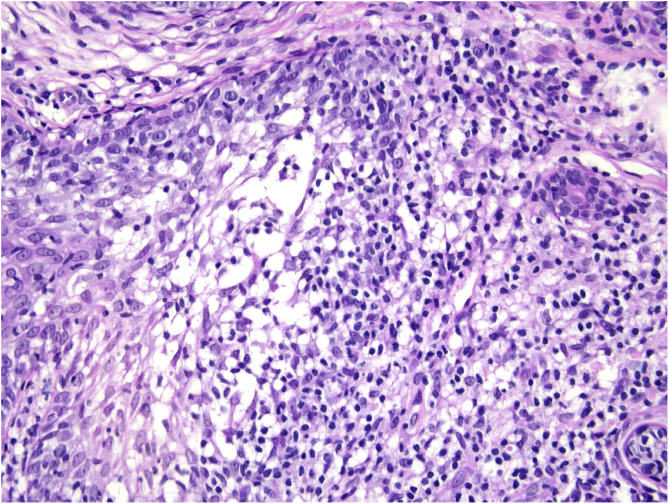
Predominantly mononuclear dense inflammatory infiltrates are seen (Hematoxylin & eosin, ×400).

**Figure 4:**
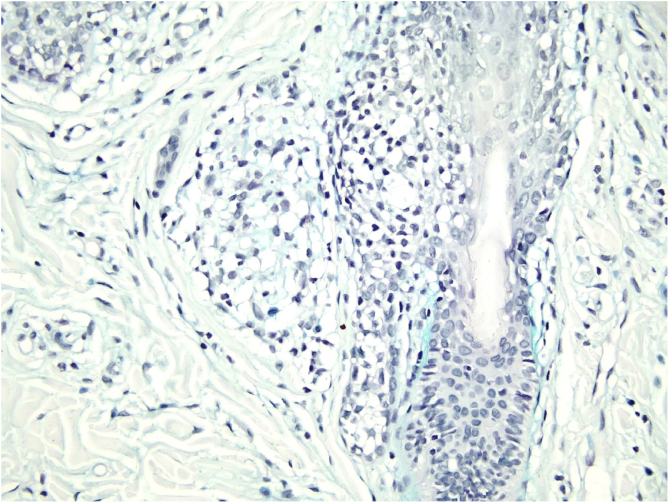
Alcian-blue-positive intercellular material possibly corresponding to mucin deposits. (Alcian blue, ×100).

**Figure 5:**
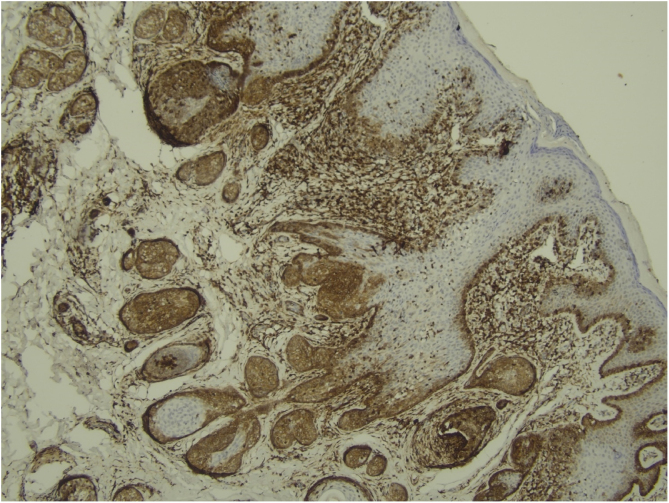
Bcl-2 positive only in the outermost tumor cells; (IMQ, 100×).

**Figure 6:**
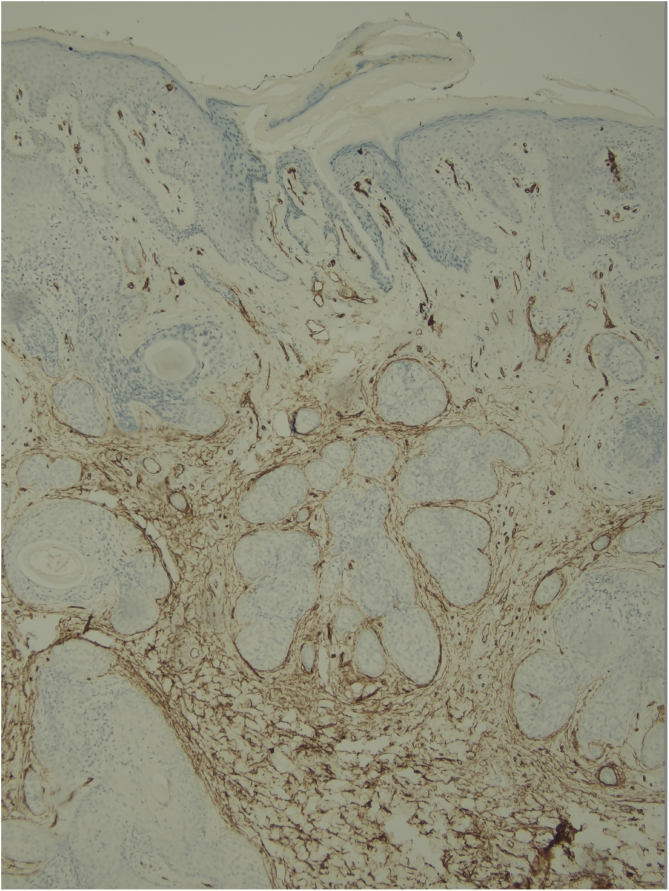
CD-34 positive in stromal tumor cells; (IMQ, 40×).

Histological examination with hematoxylin and eosin staining showed in the dermis a basaloid proliferation of basaloid cells with anastomosing cords developed from a hair follicle or lobulated nests with occasional formation of horn cysts arranged among the pilosebaceous units and fibrous stroma. Predominantly mononuclear dense inflammatory infiltrates were seen (Fig. 3). The cells of the lobulated nests were separated by Alcian-blue-positive intercellular material possibly corresponding to mucin deposits (Fig. 4). Special stains Bcl-2 demonstrated weak cytoplasmic positivity in the outermost tumor cells only (Fig. 5) and CD-34 was positive in stromal cells next to tumor cells (Fig. 6).

The lesion was surgically removed. Abdominal ultrasound, as well as panoramic maxillary and anterolateral skull XRays, were performed to rule out Gorlin-Goltz syndrome.

## Discussion

BFH is a rare, benign, superficial malformation of hair follicles that histologically presents as an epithelial proliferation of basaloid cells and clinically in various forms, with and without associated diseases.^1^ Genetic studies have linked BFH to a mutation in the PTCH-1 gene on chromosomal band 9q23. The gene is part of the same pathway involved in Gorlin-Goltz syndrome, though its expression is less severe.^1^

The PTCH-1 gene encodes a receptor for the protein product of the Sonic Hedgehog gene (SHH). The mutation of the gene determines its loss of function. The PTCH-1 receptor forms a receptor complex with another transmembrane protein known as SMO (for “smoothened”). When the SHH protein is absent, the PTCH-1 receptor inactivates SMO and keeps it from transducing a downstream signal. The binding of the SHH protein to the PTCH-1 receptor releases the suppression of SMO, causing the upregulation of hedgehog target genes through a signal cascade that involves transcription factors in the Gli family. Unregulated signaling can lead to increased cell division resulting in abnormal growth and abnormal patterning.^1^, ^2^

BFH may be congenital or acquired.^3^ The clinical presentation is diverse in the form of skin-colored-to-brown papules, nodules, or plaques on the face, scalp, and occasionally, the trunk.^1^, ^4^

Five different forms have been described: 1- solitary or multiple papules, 2- localized linear or unilateral plaque, that may be distributed along the Blaschko lines, 3- a localized plaque with alopecia, 4- generalized dominantly inherited familial type without the associated disorder, or 5- generalized papules associated with diffuse alopecia and myasthenia gravis, systemic lupus erythematosus, or cystic fibrosis.^5^, ^6^, ^7^

BFH may present alone or in association with other hereditary skin diseases, such as Bazex-Dupre-Christol syndrome, Brown-Crounse syndrome, and Happle-Tinschert syndrome.^1^, ^7^

Despite variable clinical manifestations, all types of BFH present with the same histopathological features, mainly including multifocal islands and branching cords of basaloid epithelial cells in the papillary dermis, some of which are connected to the epidermis and dilated hair follicles. The formation of keratin cysts is common within the branching cords or lace-like networks of basaloid cells. Neither cellular atypia nor mitotic figures are seen in BFH. Immunohistochemistry is positive for CD-34 in the stroma and for Bcl-2 in the periphery of the trabeculae. The Ki67 index is low.^1^, ^6^, ^7^, ^8^

The primary entity to consider in the differential diagnosis’s basal cell carcinoma (BCC), mainly the infundibulocystic subtype.^2^, ^4^, ^7^ Clinically, other differential diagnoses of localized form are: adnexal tumors, nevi, trichoepithelioma, syringocystoadenoma, intradermal melanocytic nevus, seborrheic keratosis, sebaceous nevus, syringoma, angiofibroma. BFH is a linear distribution may mimic linear epidermal nevus, striated lichen, and linear morphea. Generalized BFH may represent generalized follicular hamartoma syndrome, tuberous sclerosis, Cowden syndrome, multiple trichoepitheliomas, Gorlin-Goltz syndrome, and Rombo syndrome. The main histologic differential diagnosis includes infundibulocystic BCC, folliculocentric basaloid proliferation, and trichoepithelioma.^1^, ^6^, ^7^, ^9^

BFH should be closely monitored as they may evolve into BCC.

Different treatments, such as surgery, carbon dioxide laser, and photodynamic therapy have been proposed.^2^

Here, the authors present a rare condition with an unusual clinical and histopathological presentation that may correspond to a clinical manifestation of a mutation of the PTCH gene. To the authors’ knowledge, up to now no other cases of BFH with mucinosis and inflammation have been published in the literature.

## Financial support

None declared.

## Authors’ contributions

Solange Edelman: Drafting and editing of the manuscript; critical review of the literature; critical review of the manuscript.

Adriana Natalia Torres Huamani: Approval of the final version of the manuscript; design and planning of the study; drafting and editing of the manuscript; collection, analysis, and interpretation of data; intellectual participation in the propaedeutic and/or therapeutic conduct of the studied cases; critical review of the literature; critical review of the manuscript.

Adriana Natalia Torres Huamani: Approval of the final version of the manuscript; collection, analysis, and interpretation of data.

Andrea Bettina Cervini: Approval of the final version of the manuscript; design and planning of the study; drafting and editing of the manuscript; intellectual participation in the propaedeutic and/or therapeutic conduct of the studied cases; critical review of the literature; critical review of the manuscript.

## Conflicts of interest

None declared.
